# Surgical Outcomes of Synthetic Cartilage Implant Hemiarthroplasty for Metatarsophalangeal Arthropathy

**DOI:** 10.7759/cureus.49036

**Published:** 2023-11-19

**Authors:** Benjamin Schapira, Oscar Johnson, Radwane Faroug

**Affiliations:** 1 Trauma and Orthopedics, Lister Hospital, Stevenage, GBR

**Keywords:** metatarsophalangeal joint, hemiarthroplasty, metatarsophalangeal, polyvinyl alcohol, synthetic cartilage implant, cartiva, hallux rigidus

## Abstract

Introduction

Metatarsophalangeal joint (MTPJ) arthropathy in the great toe causes considerable pain and debilitation, severely impacting quality of life. Traditional management options included conservative measures, steroid injections, and arthrodesis. New options include Cartiva, a synthetic cartilage hemiarthroplasty for the MTPJ. This prosthesis has evidence of improved pain and function without the sacrifice of joint movement seen with arthrodesis. However, the implant itself has mixed reviews. This study aims to identify the pre-, peri, and short-term post-surgical outcomes of Cartiva surgery and review the literature for existing reported outcomes.

Methods

We retrospectively reviewed a cohort of 22 consecutive Cartiva procedures between 2016 and 2022 in a single UK institution. Hospital records were analyzed for peri-operative complications, implant survival, additional operative interventions, patient-reported outcomes, and functional improvement.

Results

Twenty-one patients underwent Cartiva for the first MTPJ pathology and one for the third MTPJ pathology. Prior to surgery, 40.9% of patients had undergone alternative therapies, including MTPJ steroid injections (seven patients), cheilectomy (four patients), and bunionectomy (one patient). Total complication rates, inclusive of medical, surgical, and implant complications were 45.5% (10/22). Total reoperation rates were 18.2% (4/22) including two revisions to arthrodesis and two manipulations under anesthesia (MUA) with local anesthetic injection. At the final follow-up, 55% were still experiencing pain, 15% neurovascular symptoms, 10% swelling, and 50% stiffness or reduced range of movement. However, 85% of patients returned to usual activities of daily living within two years.

Conclusion

Cartiva surgery for metatarsophalangeal arthropathy has demonstrated outcomes of persistent pain, limited range of movement, and restricted function at short-term follow-up. Rates of reoperation and revision to arthrodesis were comparable with similar studies.

## Introduction

Osteoarthritis of the first metatarsophalangeal joint (MTPJ) or hallux rigidus is a degenerative arthritic condition reported to affect one in six individuals over 50 years [1]. The first MTPJ demonstrates key function in normal gait, carrying up to 119% of body weight through each step [2] and as such, advanced pathology can cause severe debilitation. Low-grade or early symptomatic patients have historically benefited from non-operative interventions including activity modification, orthotics, physiotherapy, and intra-articular steroid injections [3]. However, for advanced cases, surgical options for treatment are broadly categorized into joint sparing and joint sacrificing.

Joint sparing options include cheilectomy, resurfacing, and arthroplasty. Cheilectomy involves dorsal osteophyte excision to improve pain and functional limitation driven by dorsal impingement but is often associated with persistent symptoms and high failure rates in advanced disease states [4,5]. Both MTPJ resurfacing and arthroplasty have debated success with notable reports of subsidence, osteolysis, and loosening [6] contributing to high rates of revision to arthrodesis [7,8]. In addition, failure brings greater difficulty in revision to arthrodesis due to bone loss and shortening with delayed union reported prominently following salvage surgery [6,9]. Given these outcomes, the gold standard surgical intervention for advanced Hallux Rigidus has long been considered primary arthrodesis. This joint sacrificing procedure has good outcomes for joint stability, function, and pain [10,11] but sacrifices the range of movement, footwear choice, and participation in certain sports.

In 2016, a synthetic cartilage implant (SCI), Cartiva (Cartiva Inc, Alpharetta, GA), was approved for use by the US Food and Drug Administration. Cartiva featured a cylindrical polymer hydrogel press-fit into the metatarsal head. This SCI featured good biocompatibility, a water content similar to articular cartilage, and demonstrated comparable biomechanical qualities to healthy human cartilage [12]. Cartiva offered improved pain and range of movement without sacrificing joint function. Although early industry studies reported good results [11,13], increasingly more literature has reported less satisfactory outcomes with much greater rates of further treatment, revision, and reoperation [14,15]. This study therefore aimed to evaluate the peri and short-term post-operative clinical and functional outcomes of patients undergoing Cartiva surgery in a UK District General Hospital and determine the rates of complication and reoperation. We also aimed to review the existing literature for short and mid-term outcomes following SCI surgery.

## Materials and methods

We performed a retrospective cohort study on a multisurgeon consecutive series of patients who underwent Cartiva surgery at a UK District General Hospital between 2016 and 2020 at least two years after their surgical intervention. Institutional research team approval (SE 2022032) was confirmed and as per the trust policy for retrospective cohort studies, individual participant consent was not required. Inclusion criteria were all patients over 18 years who underwent Cartiva surgery at our institution between 2016 and 2020 under the National Health Service. Cases were excluded if documentation was not available for at least two years post-surgery.

All operations were conducted by consultant foot and ankle surgeons using the standard technique recommended by the manufacturers [11] and post-operatively patients were placed in a soft dressing and post-operative shoe with wound review and removal of sutures at two weeks. Patients were advised to weight bear as tolerated with a range of movement exercises immediately and transitioned to normal footwear at two weeks. Electronic and paper medical records were analyzed for pre-operative diagnosis, symptoms and functional limitation, alternative foot, and ankle therapies prior to Cartiva, length of time between initial consult and date of surgery, peri-operative complications, and length of postoperative follow-up. Primary outcomes included implant survival and additional operative interventions with secondary outcomes including complications, patient-reported outcomes, and functional improvement. Data collection and analysis were performed by a staff member not involved with the surgery.

A thorough literature review was conducted to identify studies reporting on outcomes of Cartiva surgery and synthetic cartilage for comparison of outcomes. Search terms included “cartiva,” “synthetic cartilage implant,” “SCI,” “polyvinyl alcohol,” “metatartsophalangeal joint,” “hallux rigidus,” and “osteoarthritis” on Pubmed and Medline Ovid. Screening criteria included original studies published within the last 10 years on human subjects. Exclusion criteria included case reports, review articles, and published abstracts. Articles were appraised and analyzed formally by two authors independently for operative complications, postoperative outcomes, and reoperation rates.

Statistical analyses were conducted on Microsoft Excel Version 16.59 (Microsoft Corporation, Washington, DC, US). Descriptive statistics including average patient age, procedure length, number of outpatient appointments, and length of follow-ups were expressed as means and ranges. Total complication rates and reoperation rates were expressed as percentages (%).

## Results

In total, 24 individuals were identified, of which two had incomplete follow-ups. Therefore, 22 patients were included with 23 prostheses. Demographic information is summarized in Table 1.

**Table 1:** Patient demographic information

Patient demographic	Result		
Age	Mean 61.6 years	Range 48.2-74.2	
Male:Female ratio	1:4.5		
Laterality	11 right	10 left	1 bilateral
Length between first outpatient appointment and surgery	Mean 432.6 days	Range 38-2257	
Length of follow-up	Mean 230.9 days	Range 14-952	
Number of follow-up appointments	Mean 3.1	Range 1-9	

**Figure 1:**
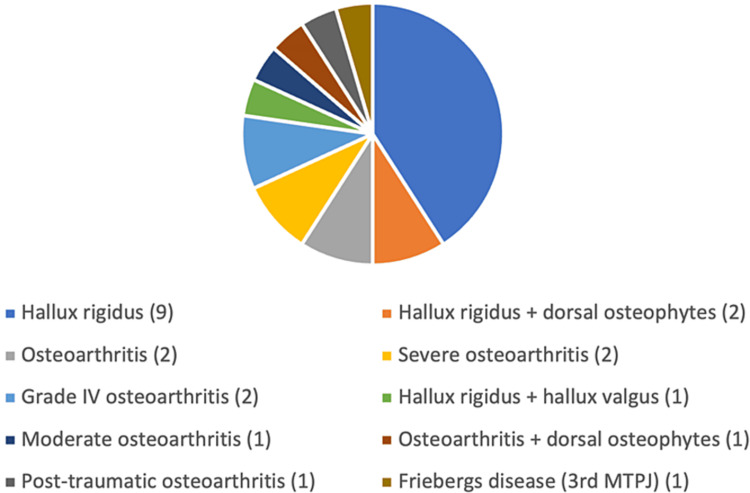
Pre-operative diagnoses as documented in outpatient clinic appointment. Number of patients in brackets

Twenty-one patients underwent Cartiva for the first MTPJ pathology and one for the third MTPJ pathology. Prior to surgery, 40.9% of patients had undergone alternative therapies, including MTPJ steroid injections (seven patients), cheilectomy (four patients), and bunionectomy (one patient). The pre-operative diagnosis was identified in all 22 patients and outlined in Figure 1.

Twenty patients were operated on under general anesthetic with or without a local anesthetic block, one patient under spinal anesthesia, and one under deep sedation. The mean length of the procedure was 50.68 minutes (range 25-93) and only one patient required admission following surgery for four days due to a new episode of atrial flutter.

Overall complication rates, inclusive of medical, surgical, and implant complications were 43.5% (10/23). Medical complications included two patients with stitch abscesses, two superficial wound infections, one oozing wound, and one patient with blistering skin. Total reoperation rates were 17.4% (4/23) including two revisions to arthrodesis and two manipulations under anesthesia (MUA) with a local anesthetic injection (both at 105 days). The first revision took place at 511 days due to reduced joint space and implant degeneration and the second at 693 days due to stress fracture of the first metatarsal, implant subsidence, and the great toe drifting into valgus. Both patients regained full weight-bearing abilities following arthrodesis. In addition, one patient was given a toe alignment splint to help with persistent symptoms. Post-operative outcomes at final follow-up recorded from outpatient documentation are expressed in Table 2.

**Table 2:** Patient-reported outcomes at final follow-up recorded from outpatient documentation after exclusion of patients who underwent revision surgery.

Patient reported outcomes	% of patients	Number of patients
Pain	55	11
Neurovascular symptoms	15	3
Swelling	10	2
Stiffness or reduced range of movement	50	10
Return to activities of daily living	85	17

In total, 61 studies reporting on Cartiva or SCI outcomes were identified. After exclusions, duplicates, and published abstracts were removed, 17 studies met all inclusion criteria for review.

## Discussion

The first MTPJ harbors complex motion, articulation, and load bearing, risking severe debilitation in advanced pathological states. This study retrospectively reviewed short-term outcomes of Cartiva surgery including patient-reported outcomes, reoperation, and revision rates. Very few studies report on Cartiva or other synthetic cartilage hemiarthroplasty prostheses and as such this study systematically reviewed all current reported outcomes and adds to the existing body of literature. The outcomes of these studies have been summarized in Tables 3, 4.

**Table 3:** A demographic review of studies reporting on outcomes of synthetic cartilage implant hemiarthroplasty for metatarsophalangeal arthropathy. Abbreviations: BMI - body mass index, M:F - male:female ratio, MTPJ - metatarsophalangeal joint, SCI - synthetic cartilage implant

Study	Type of study	Overview	Number of cases	Age	BMI	Male:female ratio	Follow-up
An et al. [15]	Retrospective observational study (only symptomatic patients).	SCI radiographic outcomes.	18 SCI.	Mean 61.3 years.	Not reported.	M:F 1:4.33.	Average 20.9 months.
Baumhauer et al. [11]	Prospective randomised multicentre, noninferiority study.	Comparison of Cartiva with arthrodesis.	130 Cartiva. 47 arthrodesis.	Mean: Cartiva 57.4 years, arthrodesis 54.9 years.	Mean Cartiva 27.2, arthrodesis 26.3.	M:F Cartiva 1:4, arthrodesis 1:3.17.	2 years.
Brandao et al. [16]	Observational study.	Comparison of Cartiva with arthrodesis.	30 Cartiva. 42 arthrodesis.	Mean: Cartiva 57 (39-80), arthrodesis 64 (41-77).	Not reported.	M:F: Cartiva 1:3.29, arthrodesis 1:1.8.	18 months (range 12-30).
Brandao et al. [17]	Observational study.	Cartiva surgery outcomes.	55 Cartiva.	Not reported.	Not reported.	M:F 1:2.92.	18 months (range 12-30).
Cassinelli et al. [14]	Retrospective observational study.	SCI surgery outcomes.	64 SCI.	Mean 62 (range 38-86).	Not reported.	M:F 1:7.	15.2 months.
Chrea et al. [18]	Retrospective comparative study.	Comparison: SCI + cheilectomy + Moberg osteotomy with cheilectomy + Moberg osteotomy.	60 SCI. 73 cheilectomy.	Mean: SCI 57 years (range 26-75), non-SCI 54 years (range 25-73).	SCI 26.6 (range 18.2-42.3), non-SCI 24.9 (range 18.2-35.4).	M:F: SCI 1:2.75, non-SCI 1:2.65.	15.6 months.
Daniels et al. [19]	Prospective observational study.	Cartiva outcomes - 5-year outcomes from Baumhauer et al. study population.	27 Cartiva.	Mean 56.1 (40.1-71.9).	27.1 (range 19.2-36.8).	M:F 1:3.5.	5.4 years.
Eble et al. [20]	Retrospective observational study.	SCI surgery outcomes.	103 SCI.	Mean 57.7 (range 26-76).	25.9 (range 18.2-42.3).	M:F 1:2.55.	26.2 months (range 14-36).
Engasser et al. [21]	Retrospective observational study.	SCI surgery outcomes.	59 SCI.	Mean 57.6 (range 39-78).	26.7 (range 18.7-35.2).	M:F 1:2.17.	18.9 months (range 3-21.3)
Glazebrook et al. [13]	Retrospective comparative study.	Comparison of Cartiva with arthrodesis.	130 Cartiva. 50 arthrodesis.	Not reported.	Not reported.	Not reported.	Not reported.
Glazebrook et al. [22]	Retrospective observational study.	Cartiva outcomes - 5-year outcomes from Baumhauer et al. study population.	112 Cartiva.	Mean 58.2 years (±8.8).	27.0 (±4.2).	M:F 1:3.48.	5.8 years (+/- 0.7).
Hoskins et al. [4]	Retrospective observational study.	Comparison of Cartiva with cheilectomy.	21 SCI, 31 cheilectomy.	Mean: Cartiva 54 (range 44-73), cheilectomy 57 (43-75).	Cartiva 25.6, cheilectomy 25.2.	M:F: Cartiva 1:9.5, cheilectomy 1:2.1.	Cartiva 30 months, cheilectomy 67 months.
Joo et al. [23]	Retrospective observational study.	Comparison of SCI with arthrodesis.	59 SCI, 122 arthrodesis. At final follow-up: 40 SCI, 61 arthrodesis.	Mean: SCI 57.5 (range 32-76), arthrodesis 61.5 (range 41-82).	Not reported.	M:F: SCI 1:2.47, cheilectomy 1:4.1.	Cartiva 27 (14-40), arthrodesis 38 (15-59).
Kilmartin et al. [24]	Observational study.	SCI outcomes (second MTPJ).	23 SCI.	Mean 55 (range 20-73).	Not reported.	M:F 1:10.	43 months (range 28-79).
Lee et al. [25]	Retrospective observational study.	SCI surgery outcomes.	96 implants in 90 patients.	Mean 54.4 (range 27-74).	25.1 (range 19.4-37.6).	M:F 1:7.	SCI 27 months (range 14-40), arthrodesis 38 months (range 15-59).
Shimozono et al. [26]	Retrospective observational study.	SCI surgery outcomes.	11 SCI.	Mean 60.3 (range 50-70).	BMI 25.4 (range 20.6-29.5).	M:F 1:1.2.	20.9 months (range 14-27).
Zanzinger et al. [27]	Retrospective case series.	SCI surgery outcomes.	44 SCI.	Mean age 55.4.	Not reported.	M:F 1:1.75.	12 months (range 10-15).

**Table 4:** A review of outcomes from studies reporting on synthetic cartilage implant hemiarthroplasty for metatarsophalangeal arthropathy. Abbreviations: ADL - activities of daily living, AOFAS - American Orthopaedic Foot & Ankle Society, EFAS - European Foot & Ankle Society, EQ-5D - Euroquol-5D, FAAM - Foot & Ankle Ability Measure, FAOS - Foot & Ankle Outcome Score, LA - local anaesthetic, MOXFQ - Manchester Oxford Foot Questionnaire, MRI - magnetic resonance imaging, MTPJ - metatarsophalangeal joint, MUA - manipulation under anaesthesia, ORIF - open reduction & internal fixation, PROMIS - Patient Reported Outcome Measurement Information System, ROM - range of movement, SCI - synthetic cartilage implant, SF-36 - short form 36, VAS - visual analogue scale, VR-12 - Veterans RAND 12 item Health Survey

Study	Complications	Outcomes	Functional scores	Range of movement	Pain	Reoperation rates	Satisfaction
An et al. [15]	Not reported.	Average loss of joint space: medial 2.00mm (p<0.001), lateral 1.6mm (p<0.001). MRI: periprosthetic oedema 100%, periprosthetic fluid 77.8%. Baseline all prostheses 10mm: post-surgery diameter 9.7mm (0.4), bony channel diameter 11.2mm (0.8) (p<0.001), implant height 9.5mm (0.6), bony channel 9.7mm (1.2).	PROMIS physical function 41 (range 27-56) (moderate physical dysfunction).	Not reported.	PROMIS pain interference 63 (range 50-74) (moderate level of pain interference).	Revision of SCI (± bone graft/Moberg osteotomy) (3) (mean 15 months). Revision to arthrodesis (2) (mean 18 months). Adhesiolysis (± MUA/Moberg osteotomy) (1) (6 and 12 months).	Not reported.
Baumhauer et al. [11]	Not reported	Any adverse events: Cartiva 69.1%, arthrodesis 72% (p=0.727). Treatment emergent events: Cartiva 44.1%, arthrodesis 42.0% (p=0.87). Serious adverse events: Cartiva 19.7%, arthrodesis 18.0% (p=0.999). Significant non inferiority of Cartiva to arthrodesis.	Cartiva scores significantly improved from baseline in SF-36, FAAM sports and ADLs. Cartiva significantly higher SF-36, FAAM sport and ADL scores up to 6 weeks post-surgery. Same trend with SF-36 PF.	Cartiva improved 6.2 degrees (27.3%) dorsiflexion - maintained at 2 years.	Cartiva: significantly improved VAS over time. At 6 weeks, 3/6 months, 1/2 years Cartiva had significantly higher VAS scores.	Cartiva: total reoperation 11.2% - revisions to arthrodesis (14) (due to persistent pain of unknown cause, mean time 390 days (54-737). MUA (1), debridement and implant repositioning (1), Moberg osteotomy of proximal phalanx (1). Arthrodesis: total reoperation 14% - hardware removal.	93% advised would have Cartiva again.
Brandao et al. [16]	Not reported.	Not reported.	FAAM sports scores: Cartiva 76.4%, arthrodesis 80.9% (p>0.3). Cartiva: 63% walking, 26% gym sports, 11% running. Arthrodesis 48% gym sports, 43% walking, 9% running.	Not reported.	Not reported.	Cartiva: 1 revision to arthrodesis (due to pain, 17 months). Arthrodesis: no reoperations.	Not reported.
Brandao et al. [17]	No complications reported.	Not reported.	FAAM ADL: baseline 64%, post-surgery 87% (p<0.0001). MOXFQ: average scores of walking/standing, pain and social interaction - baseline 58, post-surgery 34 (p<0.0001).	Not reported.	Not reported.	Cartiva-to-Cartiva revision (1) (14 months). Revision to arthrodesis (1) (17 months). MUA + steroid/LA injection (15) (12 weeks - due to stiffness)	89.4% patient satisfaction.
Cassinelli et al. [14]	Not reported.	19/64 had MRI due to continued pain/inflammation showing bone marrow oedema proximal phalanx, capsular inflammation, degeneration of metatarsosesamoid articulation.	PROMIS physical function: 42 (SD 9) - mild dysfunction.	Patient reported restricted ROM 14% - required dynamic splinting.	PROMIS pain interference 60 (SD 7) - mild pain interference	Conversion to arthrodesis: 5% (5) (average 16.4 months range 10-26 - for subsidence, persistent pain and inflammation). Implant exchange with bone graft (3) (average 12.3 months range 5-17 - for soft tissue impingement or subsidence). Adhesiolysis (4). Moberg osteotomy (1). Steroid injection: 52% ≥2 months, (mean time 7.6 months, range 2-25 and 82% ≤12 months).	Satisfaction: 14% very satisfied, 28% satisfied, 20% neutral, 11% unsatisfied, 27% very unsatisfied. 66% would have SCI again. 11% would have rather had arthrodesis.
Chrea et al. [18]	Persistent pain: SCI 11.7%, non-SCI 11.0% (p=0.9) (all managed with steroid injections, orthotics, shockwave therapy). Infection requiring antibiotics: SCI 5%.	Not reported.	PROMIS: physical function: both groups improved significantly (p<0.01). Non-SCI group significantly greater post-operative scores (p=0.043). Global physical health: both groups improved significantly (p<0.01). No significant difference between post-operative scores in each group (p=0.216).	Not reported.	PROMIS: pain intensity - both groups significantly improved post-operative scores (p<0.01). Non-SCI group significantly lower score (p=0.019). Pain interference: both groups significantly improved post-operative score (p<0.01). No significant difference between post-operative scores in both groups (p=0.248).	SCI reoperations: revision to arthrodesis (1) (14 months, inflammation and loosening). Revision hemiarthroplasty (1) (22 months - implant wear). Revision to correct hyperdorsiflexion (1) (33 months).	Not reported.
Daniels et al. [19]	Not reported	Radiographic follow-up for 85.2% of patients: no signs of loosening, subsidence, position change or wear. Proximal phalanx cysts (2), osteophytes (8).	FAAM ADL: baseline 61.4, 5-year follow-up 95.3 (p<0.001), post-operative level of function 92.6% (range 50-100). FAAM sports: baseline 39.3, 5-year follow-up 89.4 (p<0.001), post-operative level of function 92.3% (range 50-100). SF-36: baseline 39.5, post-surgery 52.2 (p<0.001). subjective function rating: 65% normal, 31% near normal, 4% abnormal.	Active dorsiflexion: baseline 9.4 (range 0-25), post-surgery 18.2 (10-30) (p<0.001). Peak dorsiflexion: baseline 20.9 (range 0-50), post-surgery 29.7 (10-45) (p<0.02).	VAS pain score: baseline 64.1, 5-year follow-up 5.7 (p<0.001).	Revision to arthrodesis (1) (2 years post-surgery due to persistent pain).	Wellbeing improvement: 69% strongly agreed, 30% agreed. 96% would undergo Cartiva again.
Eble et al. [20]	Persistent pain requiring MRI investigation (21.4%). Findings included persistent periprosthetic oedema. Non-surgical wound complications (2.9%).	Not reported.	PROMIS physical function: baseline 44.7, post-surgery 48.2 (p=0.009). PROMIS global physical health: baseline 46.9, post-surgery 50.8 (p=0.006).	Not reported.	PROMIS pain interference: baseline 58, post-surgery 52.5 (p<0.0001). PROMIS pain intensity: baseline 50.9, post-surgery 43.5 (p<0.0001).	Revision to arthrodesis (1) (14 months - inflammation and loose). Revision to a new SCI (1) (21 months - implant wear). Steroid injection 5.8% (2-11 months). 5.8% orthotics (3-6 months). ORIF for intra-operative fracture (1).	Not reported.
Engasser et al. [21]	Not reported.	Not reported.	FAAM sport: baseline 44.6, post-surgery 71 (p<0.01). FAAM ADL: baseline 71.0, post-surgery 88.2 (p<0.01). VR-12 Physical: baseline 43.4, post-surgery 49.9 (p<0.01).	Not reported.	VAS pain score: baseline 49.4, post-surgery 31.0 (p<0.01).	Total reoperations 16.9%. Revision to arthrodesis (7). Revision to arthroplasty (1). Explantation due to infection (2).	37.3% would definitely have SCI again, 35.3% would probably have SCI surgery again. 84.8% were overall satisfied.
Glazebrook et al. [13]	Not reported.	Operation time: Cartiva 35 minutes (±12.3), arthrodesis 58 minutes (±21.5) (p<0.001). Anaesthesia time: Cartiva 67 minutes (±27.8), arthrodesis 95 minutes (±41.1).	FAAM sport/ADL: significantly lesser decline in function at 2 weeks post-surgery in Cartiva group (p=0.0003/0.021). Significantly greater score in Cartiva group at 6 weeks follow up (p<0.0001/0.008) - indicating less difficult recovery period.	Not reported.	Not reported.	Not reported.	Not reported.
Glazebrook et al. [22]	Serious devise related adverse events (pain) (5).	Not reported.	FAAM ADL improved 33 (+/-17.6) from baseline (p<0.01). FAAM sports improved 47.9 (+/-27.1) from baseline (p<0.01). Sig increase from baseline but not from 2-year follow-up.	Active MTPJ peak dorsiflexion and active natural dorsiflexion maintained at 5.8 years.	VAS improved by 57.9 (+/-18.6) from baseline (p<0.01). Significant increase from baseline but not from 2y follow-up.	Revisions (9) (8.0%). Revision to arthrodesis (8). Implant removal and debridement due to infection (1) (36 months). Survivorship accounting for Baumhauer et al. initial 2 years 84.9%	Wellbeing improvement: 56.2% strongly agreed, 31.4% agreed. 93.4% would have Cartiva again.
Hoskins et al. [4]	Not reported.	Not reported.	AOFAS: significant improvement from pre- to post-surgery in Cartiva and cheilectomy groups (p<0.05), Cartiva group significantly greater post-surgery score (p=0.045). FAOS: significant improvement from pre- to post surgery in Cartiva and cheilectomy groups (p<0.05), no significant difference between groups post-operative scores (p=0.081).	Dorsal ROM: post-surgery - Cartiva 42.6 (±8.6), cheilectomy 38.1 (±7.8), both groups significant improvement (p<0.05), Cartiva significantly greater than cheilectomy (p=0.036). Plantar ROM: post-surgery - Cartiva 19.3 (±1.8) (p=0.11), cheilectomy 19.0 (±5.2), only cheilectomy significant improvement (p<0.05), no significant difference between groups (p=0.371).	Persistent pain in 10% of Cartiva group.	no reoperations reported	Not reported.
Joo et al. [23]	No significant difference in complication rates between groups (p=0.72).	Not reported.	PROMIS physical function: SCI group significantly higher score at baseline, 2 weeks, 1/2/5/6 months and final follow-up but not at 3 and 4 months. No significant difference from baseline to final follow-up in either group.	Not reported.	PROMIS pain interference: No significant difference between groups at 2 weeks, 1/2/3/4/5 months and final follow up. SCI group significantly lower score at 6 months. SCI pain at final follow-up significantly better than baseline. Persistent pain at final follow-up: 10% SCI, 8% arthrodesis (p=0.76)	SCI: conversion to arthrodesis (2) (mean 16.5 months). Arthrodesis: revision of hardware (2) (mean 24 months, hardware failure), hardware removal (1) (22 months, due to pain).	Not reported.
Kilmartin et al. [24]	Stiffness: baseline 100% of patients, post-surgery 43% of patients. Footwear restrictions: post-surgery 28%. Subsidence 4.3%. Metatarsalgia 4.3%.	Not reported.	MOXFQ: social interaction - baseline 49, post-surgery 15 (p<0.0001). Walking/standing - baseline 54, post-surgery 13 (p<0.0001). EQ-5D no significant change pre- to post-surgery. FAAM: post-surgery 91 (range 44-100) (no pre-operative scores).	ROM: post-surgery 79 degrees (range 10-80), dorsiflexion 22 degrees (range 4-40) (no preoperative measurement).	MOXFQ: pain - baseline 59, post-surgery 15 (p<0.0001).	Revision to arthrodesis (2) (9%) (mean 14 months due to implant failure).	Satisfaction: 70% totally satisfied, 13% satisfied with reservations, 17% dissatisfied. 91.3% would undergo again.
Lee et al. [25]	Wound dehiscence (2) (dressing change only).	Improvement of symptoms: 55.2% much improved, 26% improved. Time to 'new normal': <3 months 25%, 3-6 months 17.7%, 6-12 months 34.4%, >12 months 10.4%, never reached new normal 10.4%.	Sports ability: baseline - low-impact 59.4%, mid-impact 17.7%, high-impact 15.6%. Post-surgery - low-impact 57.2%, mid-impact 18.8%, high-impact 20.8%. Change in impact levels of sport activity did not reach significance (p=0.498). PROMIS physical health: 54.2 (range 26.7-67.6).	ROM: better than before surgery 68.8%, same as before surgery 7.3%, worse than before surgery 23.9%.	VAS pain score: baseline 7.9 (range 2-10), post-surgery 1.5 (0-10) (p<0.001).	Revision to arthrodesis (2) (mean 18 months). Tibial sesamoidectomy (2) (for pain refractory to steroid injections).	75% would have SCI again, 69.8% would recommend to a friend. Satisfaction: 41.7% extremely satisfied, 32.3% satisfied, 8.3% unsatisfied, 9.4% extremely unsatisfied.
Shimozono et al. [26]	Not reported.	Radiographic outcomes: joint space narrowing 85.7%. subsidence - 60% at 4 weeks and 90% at final follow-up. Proximal phalanx erosion 40%, periprosthetic lucency 50%. Implant size change: diameter reduced by 3.8%, height reduced 9.2%.	FAOS symptoms: baseline 62.2, post-surgery 64.8 (p=0.577). FAOS daily activities: baseline 81.1, post-surgery 86.1 (p=0.390). FAOS sports activities: baseline 60.8, post-surgery 62.5 (p=0.857). FAOS quality of life: baseline 42.9, post-surgery 45.5 (p=0.813).	Limited ROM (3).	VAS pain score: baseline 4.1, post-surgery 3.0 (p=0.012). FAOS pain: baseline 66.7, post-surgery 68.7 (p=0.723). Persistent pain (6).	Arthroscopic debridement and implant fixation with fibrin glue (3) (mean 12.6 months).	Satisfaction: very satisfied 18.2%, satisfied 18.2%, neutral 27.3%, unsatisfied 27.3%, very unsatisfied 9.1%.
Zanzinger et al. [27]	Not reported.	Subjective improvement: 64% slightly or much improved at final follow-up.	EFAS score: baseline 11.5, post-surgery 17.0 (p<0.05). Clinically significant improvement (>30%) in 57%. AOFAS score: baseline 58, post-surgery 75.2 (p<0.05). Clinically significant improvement (>30%) in 48%.	MTPJ ROM: baseline 31 degrees, post-surgery 32 degrees, no significant difference.	VAS pain score: baseline 6.7, post-surgery 3.5 (p<0.05). Clinically significant improvement (>30%) in 61%.	Not reported.	73% would choose Cartiva again.

Cartiva may be compared with alternative therapies for first MTPJ arthropathy including cheilectomy and arthrodesis. Regarding cheilectomy, the literature is largely split. Studies demonstrate significantly greater post-operative AOFAS functional scores and reduced rates of persistent postoperative pain in patients receiving SCI compared with cheilectomy [4]. However, converse studies demonstrate significantly greater function and pain PROMIS scores and reduced rates of postoperative infection in patients receiving cheilectomy [18]. Ultimately, both procedures present similar outcomes with no significant difference in FAOS functional scores or need for additional conservative treatments, with no differentiation of outcomes of either intervention for grades of disease severity [4,18].

Arthrodesis too presents mixed results when compared with SCI. The literature reports SCI procedures cause significantly less post-operative pain and significantly higher functional scores in the first six months post-surgery [11,23], with significantly reduced operative time and need for anesthesia [13], demonstrating a possibly faster initial recovery. However, these short-term benefits are found to reach no further significant difference at later follow-up (one and two years) [11,13,23] with evidence of significantly higher VAS pain scores in patients receiving an SCI [11]. The difference in rates of complication and adverse events requiring further treatment do not reach significance [11,23]. Interestingly, Brandao et al. reported no significant difference in FAAM sport scores and advised varying return to leisure activities, with patients undergoing arthrodesis more commonly returning to gym exercises, patients undergoing SCI more commonly returning to walking exercise and similar numbers returning to running [16], despite the known limitations on ROM following arthrodesis.

This study found a large cohort of participants to experience persistent neurovascular symptoms, swelling, and stiffness at the final follow-up. Only one study reported these symptoms, finding 43% of patients undergoing SCI to experience stiffness [24]. In this study, 50% of participants reported a restricted ROM at the final follow-up. The literature reports a varied range of post-operative ROM with short-term studies demonstrating a mean 27.3% improvement in dorsiflexion at two years [11], with a further significant increase in active and peak dorsiflexion at later follow-up [4,19]. However, Lee et al. reported mixed results with 68.8% experiencing better post-operative ROM compared to 24% experiencing a worsening ROM [25]. The number of patients reporting functional restrictions and limited ROM at final follow-up varied from 9.5% [24] to 27.3% [26] with some studies demonstrating no significant improvement in pre- to post-operative ROM [27] and reported gain only in dorsiflexion with no benefit to plantarflexion [4].

Functional assessment varied between studies in their use of different rating scales (Table 4). At both short and mid-term follow-ups, there is strong evidence to demonstrate a significant improvement in physical function, abilities in activities of daily living, walking, standing, sports, and social interaction [4,11,13,17,18,20,24,27] with one study presenting 96% of patients reaching normal or nearly normal levels of function at five years follow-up [19]. Conversely, An et al. reported their PROMIS scores at final follow-up to equate to a moderate physical dysfunction [15] with others demonstrating no significant difference in sporting abilities or PROMIS physical function scores from baseline to final follow-up [23,25]. The lack of homogeneity in rating scales makes interstudy comparisons difficult. This study did not use any patient rating scales for functional assessment but found that 85% of participants returned to their usual activities of daily living within two years of surgery.

This study found that 55% of patients were still experiencing pain at the final follow-up. The success of SCI surgery in reducing pain from baseline is well founded with reports of significant improvement in PROMIS pain interference/intensity [20], VAS [11,21,25], and MOXFQ [24] scores from as early as six weeks post-surgery. However, despite these significant improvements, a plethora of studies still report patients experience persistent pain [26]. Chrea et al. reported significantly improved PROMIS pain scores but 11.7% of participants experienced persistent postoperative pain [18] and Joo et al. found a similar trend with 10% still experiencing significant pain at the final follow-up [23]. The rates of persistent post-operative pain requiring further investigation have reached as high as 29.7% [14] with others reporting this persistence to moderately interfere with activities of daily living [15].

As a result, additional therapies have been utilized, including orthotics [20], debridement with MUA [15], and implant repositioning [11,26]. Studies report up to 27.3% of patients receive an MUA with intra-articular injection within a mean of three months post-surgery [17] which increased as high as 82% within one year [14]. This compared with our study where two patients received MUA with intra-articular injection at 105 days and one patient received a toe alignment splint. Patient satisfaction ratings ranged broadly from 66% [14] to 96% [19] informing them they would undergo SCI surgery again with most studies advising more than 80% of participants had reported improvement in their symptoms or well-being [19,22,25] (Table 4). Despite these findings, Shimozono et al. found only 36.4% of patients were satisfied [26] and Casinelli et al. found 27% to be unsatisfied [14]. Reasons commonly reported for dissatisfaction included cosmesis, persistence of symptoms, prolonged footwear restrictions, and later need for revision to arthrodesis [24].

In this study, we reported a total of six complications including stitch abscesses, superficial infections, oozing wounds, and skin blistering. Comparable studies described between 2.1% [25] and 2.9% [20] of wound complications with Chrea et al. reporting a 5% infection rate requiring antibiotics [18]. Baumhauer et al. described a complication rate of 69.1% and a serious adverse event rate of 19.7%, of which 41.6% and 45.9% required further management respectively [11]. A report on the MAUDE database found the most common cause for adverse events related to Cartiva was subsidence [8,28]. These findings were corroborated by radiographic studies demonstrating subsidence in 60% of patients as early as four weeks post-surgery and up to 90% at final follow-up [26] with subsidence identified as the cause of failure in as many as 78.3% of cases by another study [29]. Factors contributing to subsidence included a decreasing implant size [26] as well as periprosthetic swelling and edema causing osteolysis and implant loosening [14,15,20,26]. Other radiographic findings included loss of joint space [15], periarticular erosions [26], cystic changes, and osteophytes [19,22].

We found a total revision rate to arthrodesis of 8.7% due to reduced joint space with implant degeneration and a stress fracture causing subsidence and valgus drift. The short-term revision rate varied considerably in the literature from 1.9% [20] to 16.9% [21] within two years, increasing to 27.8% when considering only symptomatic patients (Table 4) [15]. In the largest multicenter randomized controlled trial, Baumhauer et al. reported a revision rate of 9.2% within two years due to persistent pain of unknown cause [11]. Amongst patients followed up for two to five years, this group experienced a further 8.0% revision due to persistent pain and infection [22]. Casinelli et al. experienced a 7.8% revision rate to arthrodesis at a mean of 16.4 months due to subsidence, persistent pain, and inflammation [14] and Chrea et al. 5% at a mean of 23 months due to loosening, implant wear, and persistent pain and hyperdorsiflexion [18]. Other reasons for revision included hardware failure with subsequent removal or revision to another SCI prosthesis [23]. Although Cartiva is considered to reduce bone damage and afford easier revision to arthrodesis compared with other joint-sparing procedures [22], there is little evidence describing the challenge of conversion and its outcomes and thus further evidence is required to substantiate this benefit.

Limitations of this study included its retrospective design and small sample size. Follow-up was available in the short term and the effects of COVID-19 were likely to confound the frequency of outpatient review. Patient-reported outcome measures and ROM were not measured preoperatively and were reported without standardized rating scales causing difficulty in direct comparison with other studies. Although Cartiva installation was documented as per the manufacturer guidelines, this multisurgeon series would have been subject to differences in technical factors that could not be accounted for. Patients had received alternative prior therapies which could have affected the outcomes related to Cartiva specifically, as demonstrated by Eble et al. with significantly greater pain intensity scores in patients with procedures prior to SCI [20]. Finally, the diagnoses documented pre-operatively were not standardized. It is unclear from these documents who would have benefited most from Cartiva surgery. The evidence fails to show a significant difference in the benefit of SCI surgery based on Coughlin and Shurnass grade [27] with other studies demonstrating no difference in benefit between Cartiva and arthrodesis regarding hallus rigidus or hallux valgus severity, gender, age, BMI, symptom duration, prior procedures or intensity of pre-operative pain [30]. The consensus as to who would benefit the most from SCI surgery is still debated and further clarification is required.

## Conclusions

In conclusion, Cartiva surgery for hallux rigidus and metatarsophalangeal arthropathy has demonstrated outcomes of persistent pain, limited range of movement, and restricted function at short-term follow-up. Rates of reoperation and revision to arthrodesis were comparable with similar studies. Future studies should focus on larger sample sizes with longer follow-ups to ascertain the optimal surgical candidate as well as long-term functional, clinical, and implant-related outcomes.
